# Comparative analyses of three complete *Primula* mitogenomes with insights into mitogenome size variation in Ericales

**DOI:** 10.1186/s12864-022-08983-x

**Published:** 2022-11-24

**Authors:** Lei Wei, Tong-Jian Liu, Gang Hao, Xue-Jun Ge, Hai-Fei Yan

**Affiliations:** 1 Key Laboratory of Plant Resources Conservation and Sustainable Utilization, South China Botanical Garden, Chinese Academy of Sciences, Guangzhou, China; 2 University of Chinese Academy of Sciences, Beijing, China; 3 College of Life Sciences, South China Agricultural University, Guangzhou, China

**Keywords:** Genome skimming, Intracellular gene transfer event, Mitochondrial genome, Phylogenetic relationship, Primulaceae

## Abstract

**Background:**

Although knowledge of the sizes, contents, and forms of plant mitochondrial genomes (mitogenomes) is increasing, little is known about the mechanisms underlying their structural diversity. Evolutionary information on the mitogenomes of *Primula*, an important ornamental taxon, is more limited than the information on their nuclear and plastid counterparts, which has hindered the comprehensive understanding of *Primula* mitogenomic diversity and evolution. The present study reported and compared three *Primula* mitogenomes and discussed the size expansion of mitogenomes in Ericales.

**Results:**

Mitogenome master circles were sequenced and successfully assembled for three *Primula* taxa and were compared with publicly available Ericales mitogenomes. The three mitogenomes contained similar gene contents and varied primarily in their structures. The *Primula* mitogenomes possessed relatively high nucleotide diversity among all examined plant lineages. In addition, high nucleotide diversity was found among *Primula* species between the Mediterranean and Himalaya-Hengduan Mountains. Most predicted RNA editing sites appeared in the second amino acid codon, increasing the hydrophobic character of the protein. An early stop in *atp6* caused by RNA editing was conserved across all examined Ericales species. The interfamilial relationships within Ericales and interspecific relationships within *Primula* could be well resolved based on mitochondrial data. Transfer of the two longest mitochondrial plastid sequences (MTPTs) occurred before the divergence of *Primula* and its close relatives, and multiple independent transfers could also occur in a single MTPT sequence. Foreign sequence [MTPTs and mitochondrial nuclear DNA sequences (NUMTs)] uptake and repeats were to some extent associated with changes in Ericales mitogenome size, although none of these relationships were significant overall.

**Conclusions:**

The present study revealed relatively conserved gene contents, gene clusters, RNA editing, and MTPTs but considerable structural variation in *Primula* mitogenomes. Relatively high nucleotide diversity was found in the *Primula* mitogenomes. In addition, mitogenomic genes, collinear gene clusters, and locally collinear blocks (LCBs) all showed phylogenetic signals. The evolutionary history of MTPTs in *Primula* was complicated, even in a single MTPT sequence. Various reasons for the size variation observed in Ericales mitogenomes were found.

**Supplementary Information:**

The online version contains supplementary material available at 10.1186/s12864-022-08983-x.

## Background

The mitochondrion is one of three genetic compartments in plant cells that play a fundamental role in encoding several essential mitochondrial electron transfer chains [1, 2]. Only 321 plant mitochondrial genomes (mitogenomes) have been released in the NCBI organelle database (organelle genome resources), while more than 7000 plant plastid genomes have been assembled and published to date (accessed on Jan. 18, 2022). Advancements in sequencing technologies and subsequently applied assembly algorithms have resulted in abundant plant mitogenomes (especially for closely related plants), which could contribute to revealing the evolutionary mechanisms of mitogenomes [3] and the domestication history of plants [4].

Plant mitogenomes vary in size but show conserved gene contents. For example, a core set of 24 protein-coding genes are generally shared among angiosperm mitogenomes [5], whereas the mitogenome length varies from 66 kb in *Viscum scurruloideum* Barlow [6] to 11.3 Mb in *Silene conica* L. [7]. RNA editing can result in amino acid sequences that differ from the corresponding conserved gene templates, which is often pervasive in the mitochondria of diverse eukaryotes (especially in land plants, [8–10]) and frequently causes the conversion of cytosine to uridine [8, 9, 11]. Another essential characteristic of plant mitogenomes is the great variation in their structures and noncoding contents [1, 5, 12]. Plant mitogenomes contain orders of magnitude more noncoding nucleotides (including introns, repetitive elements, and foreign DNA) than their metazoan counterparts [13], which contribute to the large size or the size variation of plant mtDNA genomes. First, mitochondrial introns in vascular plants play essential roles in the regulation of mitochondrial genes, which can reach 11.4 kb in *Nymphaea colorata* [14]. Group II introns are particularly prevalent within plant mitogenomes; these introns form a lariat-like structure via splicing, in contrast to group I introns. Second, the presence of repeats of various sizes in plant mitogenomes may result in high rates of genome rearrangements and even promote alternative genomic forms via recombination [12]. Third, the uptake of foreign sequences from the nuclear and plastid genomes is a vital source of noncoding sequences and might contribute to angiosperm mitogenome size expansion. For example, mitochondrial plastid sequences (MTPTs), which were first identified in maize, contribute 1 to 10% of the mitochondrial genomes of higher plants [15]. The majority of MTPTs are nonfunctional, with the exception of several tRNA genes and partial genes such as *ccmC* in *Vitis vinifera* [16]. In addition, a large plant mitogenome often includes larger mitochondrial nuclear DNA sequences (NUMTs). For example, approximately 20 and 33% of mtDNA was shown to be imported from the nucleus in apple [17] and watermelon [18], respectively. In short, the exploration of these noncoding sequences is essential to reveal the evolution of plant mitogenomes.

To date, most plant mitogenomes have been reconstructed as master circles containing the complete mitochondrial gene set [1, 7, 19–21]. Several hypotheses have been proposed to explain the size and/or structural variations of plant mitogenomes, such as different DNA repair mechanisms [22] and the integration of foreign sequences [3, 23]. However, the mechanisms underlying genome size variation and rearrangements remain unclear due to the small number of publicly available mitogenomes in plants [24–26].

The genus *Primula* L. (Primulaceae, Ericales) is an important ornamental and alpine plant group comprising more than 500 species worldwide [27–29]. The genus *Primula* has experienced rapid radiation (~ 30 Mya, [30]) and is mainly distributed in two geographically distant hotspots, the Himalaya–Hengduan Mountains in Asia and the Caucasus-Alps-Pyrenees regions in Europe [29]. The mechanisms underlying mitogenome evolution remain largely unknown because of the scarcity of well-studied mitochondrial data in *Primula* compared with the continuing progress in understanding nuclear and plastid genomes [31–33]. To our knowledge, only the *Aegiceras corniculatum* (L.) Blanco mitogenome from the family Primulaceae has been assembled thus far [34]. Thus, mitogenomes of species within *Primula* should be further studied, which may facilitate the discovery of their evolutionary dynamics on different scales, such as at the genus, family, or order level.

In this study, we aimed to explore the evolutionary history of mitogenomes among three *Primula* taxa: *Primula valentiniana* Hand.-Mazz., *Primula smithiana* Craib, and *Primula palinuri* Petagna. The first two species are restricted to alpine regions of the Himalaya–Hengduan Mountains. In contrast, *P. palinuri* is endemic to southern Italy and is adapted to the Mediterranean climate. We first sequenced and assembled the complete mitogenomes and plastomes of the three *Primula* species. Sequence repeats, foreign DNA fragments, RNA editing sites, and structural variation were analyzed in the three assembled mitogenomes. We also compared the nucleotide diversity of *Primula* taxa with that of other plant lineages. In addition, we discuss the phylogenetic application of mitogenomes and the potential causes of size variation in Ericales mitogenomes. In conclusion, our study provides insights into how the mitogenome has evolved in Ericales.

## Results

### Complete mitogenome size and gene content

**Fig. 1:**
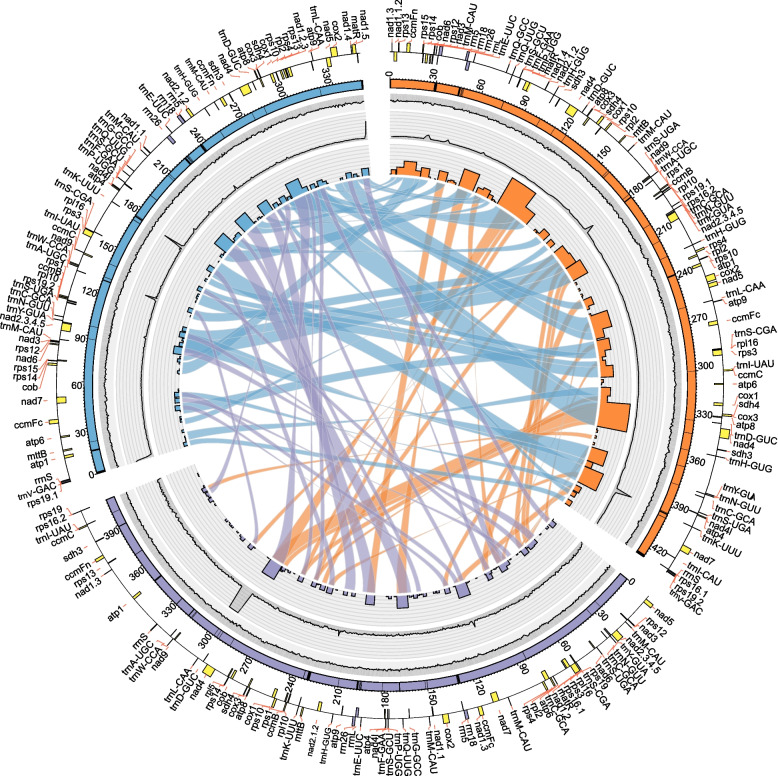
Information on the three *Primula* mitogenomes. The circular mitogenomes of *Primula smithiana* (orange), *Primula palinuri* (purple), and *Primula valentiniana* (blue) are illustrated as linear. The outer circle represents the gene and its location from outside to inside. Yellow indicates a protein-coding gene, black indicates a tRNA gene, and purple indicates a rRNA gene. The inner portion indicates mitogenome length, and the black part indicates MTPTs. The line diagrams in the inner circle represent the coverage and GC content per 500-bp window size. The innermost chord diagram represents the shared sequences between the three mitochondrial genomes, whereas the height of the histogram represents the length of each shared area

The master circles of the three *Primula* mitogenomes were successfully reconstructed using two strategies (details in the Methods). Several putative mitochondrial contigs were identified for the de novo assembly of the *P. palinuri*, *P. valentiniana*, and *P. smithiana* sequences (Table S1). Further extension and merging of these contig sequences generated complete mitogenomes with master circles of 407,597 bp, 349,360 bp, and 426,527 bp, respectively (Fig. 1; Table 1). These draft genomes were verified by complete read remapping and/or polymerase chain reaction (PCR) amplification, since all the target sequences in the merging region of raw contigs were confirmed (Fig. S1). These mitogenomes exhibited similar GC contents, ranging from 45.2 to 45.5% (Fig. 1; Table 1).

**Table 1:** General features of the three *Primula* mitogenomes

	*P. palinuri*	*P. smithiana*	*P. valentiniana*
Size	407,597 bp	426,527 bp	349,360 bp
GC content	45.5%	45.2%	45.5%
Gene numbers	61	68	64
protein coding	39	41	39
rRNA	3	3	3
tRNA	19	23	20
Introns	18	22	18
Repeat region	8105 bp	181,003 bp	4036 bp
Plastid-derived region	3073 bp	2491 bp	2857 bp
Tandem repeat patterns	8	16	7
Total length of tandem repeats	161 bp	367 bp	198 bp

Thirty-eight protein-coding genes, three rRNA genes, and 19 tRNA genes were shared among the three species (Table S2). These results suggested that the three mitogenomes presented relatively conserved gene contents. Most protein-coding genes occurred in single copies within the three mitogenomes. However, *atp8*, *cox1*, *cox3*, *nad4*, *rps10*, *sdh3*, and *sdh4* presented two gene copies in *P. smithiana* (Table S2). These mitogenomes also contained the same RNA genes (i.e., *rrn5*, *rrn18*, and *rrn26*). It was notable that although most of the 19 shared tRNA genes occurred in single copies, the *P. smithiana* mitogenome possessed two copies at the *trnC-GCA*, *trnD-GUC*, *trnM-CAU*, *trnN-GUU*, *trnS-GCU*, trnS*-UGA*, and *trnY-GUA* genes and four copies at the *trnH-GUG* gene. All *Primula* mitogenomes lacked the group I intron, whereas 30, 22, and 23 group II introns were identified in *P. smithiana*, *P. palinuri*, and *P. valentiniana*, respectively (Table 1).

### RNA editing in *Primula* mitogenomes

**Fig. 2:**
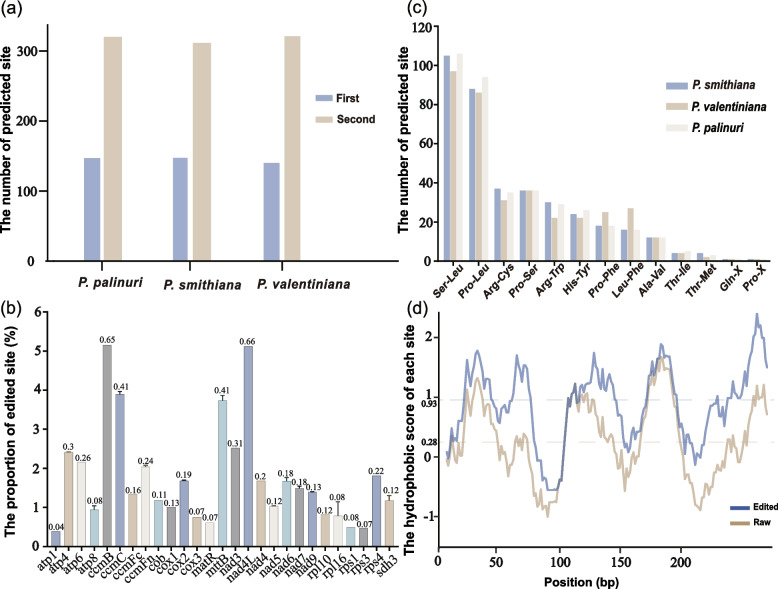
RNA editing and its potential influence. **a** Numbers of RNA editing sites in the different codons of three *Primula* mitogenomes. **b** Proportion of edited sites in the protein. The number above the bar indicates the increase in hydrophobic character after RNA editing. The trans-spliced genes are not shown. **c** Average number of amino acid changes after RNA editing. **d** Hydrophobic character of *ccmB*. The blue dotted line represents the average score, whereas the red line represents the average score of predicted proteins

The three mitogenomes exhibited similar editing site numbers predicted with the PREP-Mt server [35]. For example, *P. palinuri*, *P. valentiniana*, and *P. smithiana* contained 467, 460, and 456 C-U editing sites, respectively (Fig. 2a). Approximately 300 RNA editing sites occurred at the 2nd base of the codon in each of the three *Primula* species. In contrast, approximately 140 sites occurred at the 1st base of the codon (Fig. 2a). Although the RNA editing sites were not evenly distributed among coding genes, the hydrophobic properties of all proteins were increased after editing (Fig. 2b). These amino acid changes mainly involved Ser-Leu, Pro-Leu, Arg-Cys, Pro-Ser, and His-Tyr transitions (Fig. 2c). For example, the average hydrophobic character per site in *ccmB* increased from 0.28 to 0.93 (Fig. 2d).

Two RNA editing sites in *atp6* and *ccmFc* produced stop codons in all *Primula* taxa. An editing-derived stop codon resulted in a 107-bp deletion in *atp6* (Fig. S2), and an RNA editing site occurred at the penultimate amino acid codon in *ccmFc* (Fig. S3). Notably, both editing sites were also found across the other eight Ericales mitogenomes (Figs. S2–3). The editing efficiency of the edited-introducing stop codon in *atp6* ranged from 50% (*C. sinensis*) to 100% (*R. simsii*), suggesting effective termination. However, the editing efficiency for *ccmFc* was 0, indicating that the predicted RNA editing site was a false positive.

### Repeats and foreign-derived sequence identification

**Fig. 3:**
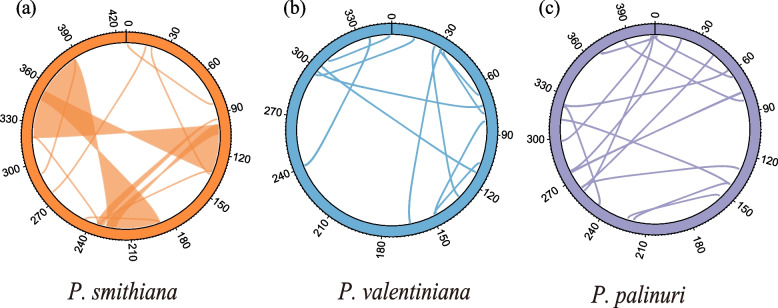
Repeat region and coordinate of the three longest shared regions among *Primula* mitogenomes. **a** Repeat sequences in *Primula smithiana* (orange). **b** *Primula valentiniana* (blue). **c** *Primula palinuri* (purple)

The total length of repeat regions in the three *Primula* mitogenomes varied dramatically. The *P. smithiana* mitogenome contained repeats with a total length of 181,003 bp (Fig. 3a), accounting for over 30% of the whole mitogenome sequence. *P. palinuri* and *P. valentiniana* exhibited relatively compact mitogenomes with total repeat lengths of 8105 bp and 4036 bp, respectively (Fig. 3b-c). The *Primula* mitogenomes lacked large tandem repeats (Tables S3–5).

Eleven MTPTs were identified within the three *Primula* taxa, and seven were commonly identified in the three species (Table S6). In addition, MTPT2 and MTPT6 were specific to *P. palinuri*, whereas MTPT8 and MTPT9 were specific to *P. smithiana* and *P. valentiniana*. Only the first two largest MTPTs (MTPT7 and MTPT10, both longer than 500 bp) were extracted for further analysis, as the *Primula* mitogenomes lacked large MTPTs. The depths of these MTPTs ranged from 44× to 80×, whereas those of their plastid counterparts ranged from 147.5× to 828.5× (Fig. S4). The presence of the two MTPTs was further verified through PCR amplification (Fig. S1). The GC contents of MTPT7 were 38.5% (*P. smithiana*), 38.7% (*P. valentiniana*), and 40.9% (*P. palinuri*), whereas those of MTPT10 were approximately 50–51% among the three species.

**Fig. 4:**
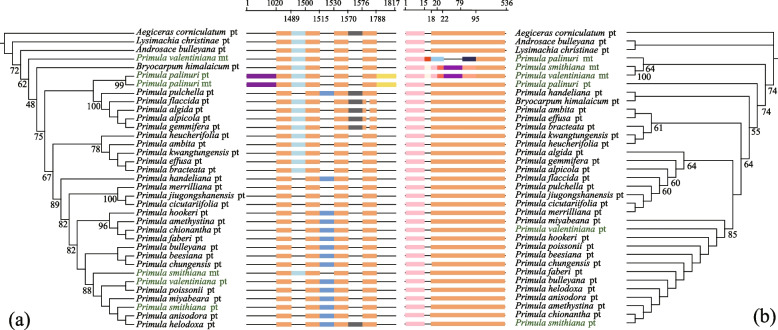
Phylogenetic relationship of MTPT7 (**a**) and MTPT10 (**b**). Bootstrap values (> 50) are shown above the branches. pt. and mt indicate plastid and mitochondrial sequences, respectively. The alignment result is shown in the middle, in which the number at the top represents the sequence site, and different colors distinguish all parts of the alignment

The phylogenetic relationships indicated that MTPT7 might have experienced three independent transfer events (Fig. 4a). One may have occurred after the speciation of *P. palinuri,* as its MTPT7 was grouped with its plastid counterpart. In contrast, the other two were distributed at distant positions in the phylogenetic tree (Fig. 4a). In particular, MTPT7 occurred very recently, with a unique insertion (approximately 1000 bp, Fig. 4a) in *P. palinuri*, exhibiting a higher GC content (40.9%). The oldest MTPT7 event occurred around the time of divergence between *Androsace* L. and *Bryocarpum* Hook. f. & Thomson (Fig. 4a), whereas MTPT10 probably appeared before the diversification of *Primula* (Fig. 4b).

NUMTs were searched against the *P. veris* L. nuclear genome (GCA_000788445.1). The total length of nuclear-shared sequences ranged from 210 kb to 266 kb. The longest nuclear-shared sequence was 9928 bp, which occurred in *P. smithiana* (Table S7).

### Locally collinear blocks and gene clusters in *Primula* mitogenomes

The locally collinear blocks (LCBs) are the conserved genomic sequences among all genomes, which is useful for mitogenome structural analysis and for constructing a robust phylogenetic topology [36]. The LCBs among the three *Primula* mitogenomes were scattered in the mitogenomes, and no specific conserved region was longer than 50 kb (Fig. 1; Fig. S5). Despite the relatively short shared regions, the total length of the shared regions was extremely long. The shared sequences between *P. smithiana* and *P. valentiniana* were over 364 kb in length, which was markedly longer than in the other species pairs [*P. smithiana* and *P. palinuri* (288 kb), and *P. valentiniana* and *P. palinuri* (243 kb), Fig. 1].

**Fig. 5:**
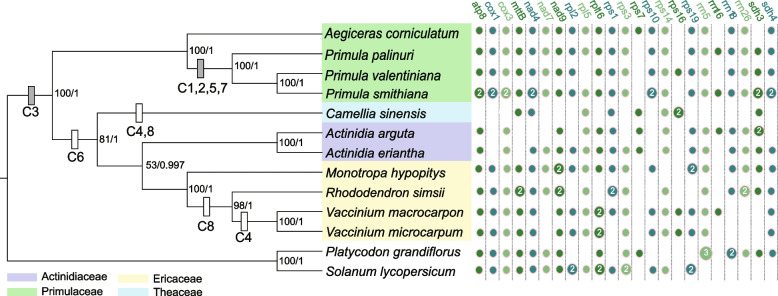
Phylogenetic relationships of 11 Ericales mitogenomes based on 13 shared protein-coding genes. The support values are illustrated on the branches (left: the ultrafast bootstrap of the ML tree; right: the posterior probability of Bayesian inference). The dot plot represents the gene content in each mitogenome; however, genes shared by all mitogenomes or only lost in one are not illustrated. The rectangle represents the eight gene clusters identified in the three *Primula* mitogenomes, with C1-C8 representing cluster 1-cluster 8 (detailed information on each cluster is illustrated in Fig. S7). White and gray rectangles represent the loss and retention of these clusters

Among the 29 conserved gene clusters identified in angiosperms [25], the three *Primula* mitogenomes possessed 13 of these shared gene clusters, whereas *P. palinuri* exhibited one additional gene cluster (*>trnI-CAT > <trnD-GTC<*) (Fig. S6). Eight additional gene clusters were also identified, four of which were specific to *Primula* compared to the other Ericales mitogenomes (Fig. 5; Fig. S7). Among the four specific gene clusters, each of the three gene clusters [*nad9-trnW* (*CGA*)*-trnA* (*UGC*) and *rps12-nad3-trnM* (*CAU*)] contained two (*trnW-CGA* and *trnA-UGC*) and one (*trnM-CAU*) plastid-derived gene, respectively.

### Nucleotide diversity based on locally collinear blocks of mitogenomes

The nucleotide diversity (π) of LCBs was calculated for different plant lineages (Table S8)*.* First, *Primula* mitogenomes exhibited relatively high nucleotide diversity (π: 0.009) among all examined plant lineages, which was 10-fold higher than the lowest diversity, found in *Zea* (π: 0.0009) (Table S8). The π value (0.005) among the CDS regions of *Primula* was also higher than that in most of the analyzed taxa (Table S8). Second, the high nucleotide diversity at the genus level mainly contributed to the striking variation between *P. palinuri* (from the Mediterranean region) and the two alpine *Primula* taxa (from the Himalaya–Hengduan Mountains). The π value between the two alpine *Primula* taxa was 0.004, while the π value between the taxa from the two regions (i.e., the Himalaya–Hengduan Mountains and the Mediterranean regions) was 0.013 (Table S9).

### Size variations and phylogenetic analyses among Ericales mitogenomes

**Fig. 6:**
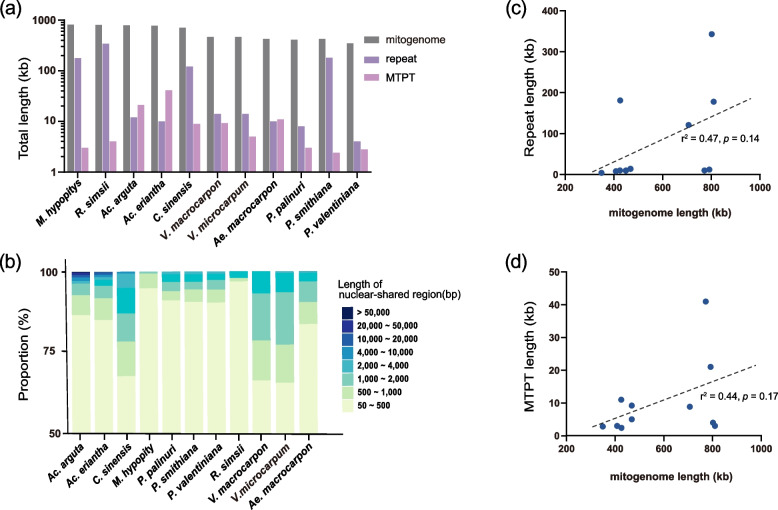
Mitogenome sizes, repeats, MTPTs within Ericales, and their correlations. **a** Total length of different Ericales mitogenome components. **b** Proportions of different lengths of nuclear-shared sequences in Ericales mitogenomes. **c** Correlation of repeat and Ericales mitogenome size. The proportion started at 50% because most nuclear-shared sequences were < 500 bp. **d** Correlation of MTPTs and Ericales mitogenome size

The present study included eight published Ericales mitogenomes (from Primulaceae, Ericaceae, Actinidiaceae, and Theaceae) from NCBI, with the aim of revealing the potential causes of the observed mitogenome size variation at the order level (Table S10). The *Monotropa hypopitys* Crantz, *Rhododendron simsii* Planch., *Actinidia argute* Miq, *Actinidia eriantha* Benth., and *Camellia sinensis* (L.) Kuntze mitogenome sizes were 810 kb, 802 kb, 792 kb, 772 kb, and 707 kb, respectively, which were much larger than those of *Vaccinium macrocarpon* Aiton (459 kb), *V. microcarpum* Miyabe & T. Miyake (468 kb), *Ae. corniculatum* (425 kb), and the three *Primula* taxa (349–426 kb) (Fig. 6a). The total repeat lengths of *R. simsii* (343 kb), *P. smithiana* (181 kb), *M. hypopitys* (178), and *C. sinensis* (121 kb) were significantly larger than those of the remaining mitogenomes (each < 20 kb) (Fig. 6a). Most Ericales mitogenomes lacked large MTPTs, whereas total lengths of 21 kb and 41 kb were identified in *Ac. arguta* and *Ac. eriantha*, respectively (Fig. 6a).

Most Ericales NUMTs were < 500 bp, and the longest NUMT was identified in *Ac. arguta* (53 kb) (Fig. 6b). Additionally, there was a positive correlation between the NUMTs and Ericales mitogenome length, although the *p* value was slightly higher than 0.05 (Fig. S8). The relatively small mitogenome of *Ae. corniculatum* contained a large number of NUMTs (Table S10), which indicated that this species served as an outlier in this analysis. There was a very strong positive relationship between NUMTs and mitogenome sizes when *Ae. corniculatum* was removed (r^2^ = 0.55, *P* = 0.01, Fig. S9a). Pearson’s correlation test also indicated that repeats (Fig. 6c) were not significantly correlated with Ericales mitogenome size variation. However, a significant correlation was observed between repeats and mitogenome size when the kiwifruit mitogenomes were removed (Fig. S9b), since the large kiwifruit mitogenomes contained a small number of repeats [3]. There was also no significant relationship between MTPTs and mitogenome sizes in Ericales (Fig. 6d). *R. simsii* and *M. hypopitys* both showed relatively large mitogenomes but fewer MTPTs (Table S10). Therefore, the two species act as outliers in this analysis. There was a very strong positive relationship between MTPTs and mitogenome sizes when *R. simsii* and *M. hypopitys* were removed (r^2^ = 0.6, *P* = 0.01, Fig. S9c).

Protein-coding genes also varied within Ericales, and only 13 of these genes were shared among all eight Ericales taxa (Fig. 5). 38 protein-coding genes were shared between *Primula* taxa and *Ae. corniculatum*, whereas only 28 and 27 genes were observed in *C. sinensis* and *Ac. arguta*, respectively.

Both maximum likelihood (ML) and Bayesian inference trees were reconstructed based on the 13 shared protein-coding genes (with a total aligned length of 11,360 bp). Both trees suggested that *P. smithiana* (Sect. *Proliferae*) and *P. valentiniana* (Sect. *Amethyatina*) exhibited the closest relationship (Fig. 5). The interfamilial relationships (i.e., Primulaceae, Ericaceae, Actinidiaceae, and Theaceae) within Ericales species with available mitogenome data to date were resolved robustly. Primulaceae is sister to other families. Among the three other families, Theaceae was sister to a clade that contained Ericaceae and Actinidiaceae.

## Discussion

### Complete mitogenomes of three *Primula* taxa

The plant mitogenome is known for its considerable variation in both structure and content [1, 5, 12]. Most mitogenomes are reconstructed as a master circle containing the complete mitochondrial gene set [1, 7, 19–21]. A previously assembled pipeline [37–39] was followed to extend and merge the *Primula* mitochondrial contigs into a master circle. These master circles indicated highly homologous recombination (HR) frequencies, as no long collinear sequences were shared within *Primula* (Fig. S5) [3]. Most potential HR events occurred in the noncoding region and seemed to occur randomly among *Primula* mitogenomes, as no synteny could be detected based on the coordinates of each shared region (Fig. S5). Large repeats (> 500 bp) are often involved in reversible reciprocal HR that modulates plant mitogenome plasticity [25, 40, 41]. However, only the *P. smithiana* mitogenome exhibited a high proportion of repeats. As mitogenome reconstruction for *P. palinuri* and *P. valentiniana* involved the merging of several contigs, long repeats (over 30 kb in *P. smithiana*, Fig. 3a) might have been consolidated and eventually “vanished” from the final assemblies. This scenario could be examined using long-read sequencing technologies in the future.

Studies have discussed the subgenomic molecular forms of plant mitogenomes from linear branches to several circular sequences [42, 43]. The master circle reconstructed in the present study might not fully represent the only state of the *Primula* mitogenomes. However, the master circle contained all the mitochondrial genetic information [20, 23, 44]. The present study implemented two methods for confirming the reliability of our master circles. First, the clean reads were remapped against the master circle to identify potential assembly mistakes. Second, several PCR primer pairs were designed to verify the accuracy of the merging of contigs. The consecutive mapping and PCR results indicated the reliability of the master circles of the *Primula* mitogenomes (Fig. S1).

### Contrasting patterns of nucleotide diversity across plant lineages

Compared with plant nuclear and plastid genomes, high rates of rearrangements in plant mitogenome frequently occur among closely related species [12]. Therefore, the conserved coding region and collinear segments might be relatively good resources for comparative analyses among different plant lineages [12, 20]. The study of mitochondrial collinear regions could further help us understand the evolution of plant mitogenomes [3, 36]. However, the lack of shared collinear fragments among plant lineages is due to frequent repeat-mediated rearrangement [12] and a lack of large collinear fragments, as shown in *Picea* [45].

In this study, we calculated and compared the nucleotide diversity of three collinear fragments of the mitogenomes within each genus (including at least three mitogenomes each). *Primula* mitogenomes exhibited the highest nucleotide diversity (Table S8), which was 2–3 times lower than the nucleotide diversity of the plastid genomes (complete sequence: 0.026; CDS: 0.019). This scenario is consistent with the synonymous-site divergence levels observed within the plastome and mitogenome [46].

There are several possible explanations for the contrasting patterns of nucleotide diversity within *Primula* and among plant lineages. First, *Primula* probably presents an elevated mutation rate in the mitogenome, as shown in several plant lineages [47]. Several functional genes might be positively selected by severe environmental conditions (such as strong ultraviolet radiation and extremely low temperatures in alpine environments) [48, 49], with far-reaching impacts on noncoding regions because of the hitchhiking effect. However, there is no clear evidence of elevated rates of mutation in these core genes. Second, a plausible alternative is that the LCB evolved under an assumed constant mutation rate, although substantial rate variation occurs among plant lineages. The specific evolutionary history of each plant genus should result in striking mitogenomic diversity. For example, the most recent common ancestor (MRCA) of the *Primula* taxa in the two regions (Europe vs. Asia) originated 20–40 Mya [30, 50], which is the oldest age among the taxa examined in this study, contributing to the accumulation of the greatest amount of nucleotide polymorphism. However, the MRCA of the two alpine *Primula* taxa in the Himalaya–Hengduan Mountains probably originated in the Late Miocene (approx*.* 9 Mya, [50]), resulting in approximately three times less nucleotide polymorphism than was observed between the two regions (Europe vs. Asia). At the other extreme, the genus *Zea*, which has diversified since approx*.* 0.18 Mya [51], presented the lowest interspecific polymorphism (Table S8). Third, the role of sampling error should be taken into account in considering these contrasting patterns of nucleotide polymorphism among plant lineages. Given that the LCB examined here accounted for a varying fraction of the whole mitogenome (4–64%), more LCB data and more comprehensive taxon sampling are required to test this in the future.

### RNA editing is essential for mitochondrial gene expression

RNA editing is a common phenomenon within land plant mitogenomes involving the conversion of cytidines to uridines (C-U editing) or uridines to cytidines (U-C editing) in some plants [11, 52]. The most predictable RNA editing sites were located on the second codon in *Primula*, similar to most angiosperm mitogenomes [53].

Most of the predicted RNA editing sites could result in the conversion of the encoded amino acid from neutral to hydrophobic, ultimately increasing the hydrophobic character of the coding protein [53]. Hydrophobicity is conducive to protein folding and secondary structure formation [54]. The most frequent RNA editing events were found in *ccmB* and *nad4L* in *P. smithiana*. The secondary and tertiary structures of these two genes in *P. smithiana* showed increased stability of the protein.

In this study, only two RNA editing sites (in the *atp6* and *ccmFc* genes) that could result in an early stop codon were identified in *Primula*, which is lower than the number found in kiwifruit mitogenomes [3]. Although both RNA editing sites could also be detected in other Ericales taxa (Figs. S2–3), only the termination in the *atp6* gene was effective. The regulation of *atp6* is essential for plant fertilization [55] and is related to cytoplasmic male sterility (CMS) in pepper [56], sunflower [57], rice [58], and *Sorghum* [59]. RNA editing in the *atp6* gene also alters seed formation in maize [60]. Therefore, this RNA editing site in the *atp6* gene may potentially impact the reproduction of Ericales species, but further studies are needed to verify this. Furthermore, our results highlight the importance of considering RNA editing sites during annotation.

### Plastid-derived sequences in *Primula* mitogenomes

Transfer events from plastid to mitochondrial genomes frequently occur in angiosperm plants [61, 62]. Studies have revealed plastid-derived backgrounds of 0.1–11.5% of plant mitogenomes [63]. The present study identified 0.5–0.7% of *Primula* mitogenomes as MTPTs (Table 1).

MTPT transfer occurs independently among species [15], and plastid-derived fragments ultimately become nonfunctional pseudogenes [63]. Sequence transfer events from plastids to mitochondria inferred based on the oldest MTPT (*trnV (uac)-trnM (cau)-atpE-atpB-rbcL*) occurred at least 300 million years ago (Mya), before the divergence of extant gymnosperms and angiosperms [64]. In the present study, the oldest MTPT transfer events (MTPT7 and MTPT10) might date back to the divergence time of *Primula* and its relatives (approximately 23–40 Mya) (Fig. 4) [30]. However, a specific transfer event (such as MTPT7) probably occurred multiple times, as reported in several plant lineages [65].

Two hypotheses may explain the multiple transfer events observed. First, these transfer events could have been acquired directly via intracellular gene transfer (IGT). An ancient plastome-to-mitogenome transfer event occurred in ancestral species of *Primula* and its relatives but was partially lost in several species during the subsequent speciation process, after which IGT reoccurred recently. Second, the transfer event was acquired by plant-to-plant horizontal gene transfer (HGT). This means that the “ancient” MTPT was initially acquired via mitochondrion-to-mitochondrion HGT among plant taxa, rather than via an ancient plastid-to-mitochondrial IGT event [66]. Regardless, the underlying mechanism of MTPT deserves in-depth study in the future.

### Phylogenetic implications from mitogenomes

Unlike plastid and nuclear genes (such as low copy nuclear genes or ITS), the mitogenome is not frequently used to reconstruct phylogenies or phylogeographies in higher plants due to its slow mutation rate [46], frequent genomic rearrangement [12], and incorporation of foreign DNA from the nuclear and plastid genomes [23]. However, several mitogenomic genes (such as *atp1*, *matR*, *nad5*, and *rps3*) have been widely used for plant phylogenetic studies at different levels since they may offer different insights than plastid and nuclear genes [67], see references therein). Theoretically, the coding genes of mitogenomes are more suitable for elucidating ancient diversification patterns in plants because of their generally slow mutation rates compared to plastid and nuclear genes [46].

In this study, the interfamilial relationships (i.e., Primulaceae, Ericaceae, Actinidiaceae, and Theaceae) within Ericales were resolved robustly based on the available mitogenome data (Fig. 5). Notably, these relationships are broadly consistent with previous studies with multiple fragment combinations (containing plastid, mitogenome, and nuclear genes [50];), plastomes [68], and transcriptomes and genomes [69, 70].

The three studied *Primula* taxa belong to two subgenera (*Aleuritia* and *Auriculastrum*) [28, 29]. Subgenus *Aleuritia* contains *P. smithiana* (Sect. *Proliferae*) and *P. valentiniana* (Sect. *Amethyatina*), whereas subgenus *Auriculastrum* includes *P. palinuri* (Sect. *Auricula*). Here, *P. palinuri* is sister to a clade that contains *P. smithiana* and *P. valentiniana*, consistent with the relationships based on plastid genes [30, 71].

The identified collinear gene clusters and LCB verified the above relationships in *Primula*. First, among 13 conserved mitochondrial gene clusters in *Primula* taxa (Fig. S6), one unique gene cluster (*trnI-CAT-trnD-GTC*) occurred only in *P. palinuri*. Second, the collinear fragments of the mitogenomes between *P. smithiana* and *P. valentiniana* exhibited lower nucleotide diversity than the other pairs containing *P. palinuri* (Table S9). The above two lines of evidence indicated the close relationship of *P. smithiana* and *P. valentiniana*. These results show that some conserved gene clusters and LCBs of plant mitogenomes present phylogenetic signals [36, 72].

### Mitogenome size expansion in Ericales

Several hypotheses have been proposed to explain the considerable variation in mitogenome size observed in land plants [3, 13, 22, 23]. However, further specific studies are required [73]. The variation in noncoding sequences probably results in size variation [74], as most angiosperm mitogenomes generally contain a core set of 24 protein-coding genes [47], and 100-fold variation is observed among total plant mitogenome sizes [5].

The uptake of foreign sequences is a vital source of noncoding sequences [17, 23, 61, 75] and might contribute to angiosperm mitogenome size expansion. A large plant mitogenome often contains longer nuclear-shared sequences [76]. NUMT seems to contribute to mitogenome size in Ericales, despite the lack of a strong overall NUMT-mitogenome size relationship (Fig. S8). MTPT is also sometimes linked to the size variation of angiosperm mitogenomes [25, 77]. However, no significant relationship between MTPTs and mitogenome sizes was found in Ericales (Fig. S6d). The relatively large mitogenomes of *R. simsii* and *M. hypopitys* do not possess large MTPTs, probably because of the lack of an inverted repeat region (IR) in *R. simsii* [78] or because all genes encode products with photosynthetic functions and RNA polymerase subunits in the mycoheterotrophic plant *M. hypopitys* [79]. Collectively, the results indicated that the uptake of foreign sequences is a vital source of mitogenome size expansion within Ericales, albeit with some exceptions.

Repeats usually cause plant mitogenome size variation [20]. Approximately 42.7% of the *R. simsii* mitogenome consisted of repeat sequences, probably resulting in the largest mitogenome in Ericales. However, an exception was observed in the kiwifruit mitogenomes (Fig. 6c), which possessed large mitogenomes but lacked repeats [3], suggesting diverse reasons for mitogenome size variation in the Ericales order. Further studies are needed to examine plant mitogenome size expansion and variation.

## Conclusions

This study successfully assembled the complete mitogenomes of three *Primula* species in the form of master circles. These mitogenomes shared similar gene contents but varied in structure. Relatively high nucleotide diversity was found in the *Primula* mitogenomes among all examined plant lineages. The RNA editing of these *Primula* mitogenomes could increase protein stability and alleviate the influence of amino acid mutation. MTPT events that co-occurred in the mitogenome and plastome were identified using the phylogenetic method, indicating that multiple transfer events probably occurred in the evolutionary history of *Primula*. In addition, mitogenomic genes, collinear gene clusters, and LCB all showed phylogenetic signals. Although the size of IGT events and repeats might drive mitogenome size variation in Ericales, the diverse reasons for mitogenome size variation in the order must be studied further.

## Methods

### Plant materials, DNA extraction, and sequencing

Fresh young leaves of *P. smithiana* (voucher no. Xu et al. 150,170) were collected from the South China Botanical Garden greenhouse, Chinese Academy of Sciences (Guangzhou, China), and cultivated from Yadong County of Tibet, China. Mitochondria were extracted from the leaves using density gradient centrifugation as described in a previous study [80]. mtDNA was extracted using the modified cetyltrimethylammonium bromide (CTAB) method [81]. Silica gel-dried leaves of *P. valentiniana* (voucher no. Hao et al. 120,274) were used for DNA extraction via the CTAB method [81]. The Kew DNA Bank provided the total DNA of *P. palinuri* (voucher no. Chase M.W. 16,567, Kew) (https://dnabank.science.kew.org). These voucher specimens were formally identified or checked by the third author (Prof. Gang Hao, South China Agricultural University) and deposited in the herbarium of the South China Botanical Garden, Chinese Academy of Sciences (IBSC). All experimental research complied with relevant institutional, national, and international guidelines and legislation. No specific permissions or licenses were required for our collection activities and experiments. A 250-bp paired-end library was prepared and sequenced on the Illumina HiSeq 2500 platform (Illumina, San Diego, CA, USA).

### Genome assembly and validation

The short reads were checked using FastQC (http://www.bioinformatics.babraham.ac.uk/projects/fastqc/) and trimmed using Trimmomatic [82] for the *P. smithiana* assembly. The clean reads were assembled de novo using Spades v3.13.0 [83], and putative contigs were then selected and extended using PRICE [84] with the following parameters: *600 95 -nc 50 -dbmax 72 -mol 30 -mpi 90 -target 90 2 1 1*.

Genome skimming data for *P. palinuri* and *P. valentiniana* were used after quality control for de novo assembly by MEGAHIT v1.0 [85] and Spades v3.13.0 [83] to maximize the utilization of paired-end reads. Furthermore, the potential mitochondrial contigs were selected based on coverage and rechecked manually by BLASTN [86] against the NCBI nonredundant nucleotide database. The whole trimmed reads were then mapped to the putative mitogenome contigs with Geneious (http://www.geneious.com/). Contigs were extended based on the mapping coverage as described by Mower et al. [87] and Zhang et al. [39]. Mapped sequences were excluded if their sequencing coverage depth was < 5× and > 500× or the overlap was < 50 bp. In the continuous extension cycle, contigs showing overlap of greater than 80 bp and identity over 99% were merged.

The complete reads were aligned against these draft mitogenomes using BWA v0.7 [67] with the default parameters to evaluate the coverage consistency and read connectivity. The sites that differed from 75% of the other aligned reads were corrected.

Although several isoforms of angiosperm mitochondrial sequences have been reported, the assembled form of a master circle containing all mitochondrial genes is commonly found in various plant groups [1, 3, 5, 42]. To further check the reliability of our assembly, five PCR primer pairs were designed between two initial contigs for each species to confirm whether these assembled regions could be successfully amplified (Fig. S1; Table S11).

### Annotation of *Primula* mitogenomes

Protein and rRNA genes were annotated using the GeSeq online server [88] and local BLASTN [86], with eight Ericales mitogenomes [*Ae. Corniculatum* (MT130509.1), *R. simsii* (MW030508.1), *C. sinensis* (NC_043914.1), *V. macrocarpon* (NC_023338.1), *V. microcarpum* (MK715445.1), *M. hypopitys,* (MK990822, MK990823), *Ac. arguta* (MH559343), and *A. eriantha* (MH645952.1)] as references. tRNAs were identified by using tRNAscan-SE v2.0.7 [89]. The start and stop codons in exons were adjusted manually. Group I and II introns were detected with the RNAweasel tool [90]. ORF-Finder was used for predicting open reading frames longer than 300 bp with the standard genetic code (https://www.ncbi.nlm.nih.gov/orffinder/). The GC count per 500 bp was calculated with an in-house Python3 script. The depth of sequencing coverage per locus was calculated using the genomecov command in bbMap (https://www.sourceforge.net/projects/bbmap/) with the parameter *–d*.

Repeat regions were identified by alignment against each assembly using BLASTN [86] with a minimum identity of 85% and a minimum length of 100 bp. Tandem repeat sequences were detected by using Tandem Repeats Finder [91] with the default parameters. The detailed information of the three *Primula* mitogenomes was visualized using Circos v0.69 [92].

### Identification of RNA editing sites and gene clusters

RNA editing sites in the three mitogenomes were predicted using the PRET-Mt server [35] with a cutoff value of 0.2. The hydrophobicity of *ccmB* and *nad4L* in *P. smithiana* were calculated using ProtScale [93]. In contrast, the average hydrophobic character of *P. smithiana* protein-coding genes was calculated based on the method described by Kyte and Doolittle [94].

The *atp6* and *ccmFc* gene sequences were extracted from the eight publicly available Ericales and *Primula* mitogenomes and were aligned using MAFFT v.7.4 [95]. The secondary structures of *atp6* and *ccmFc* were inferred using PSIPRED [96]. To validate the predicted editing sites, the RNA-seq data of *Ac. arguta* (SRR3823655), *Ae. Corniculatum* (SRR1688722), *C. sinensis* (SRR20083852), *M. hypopitys* (SRR10159707), *R. simsii* (SRR10415549), and *V. macrocarpon* (SRR18449568) were downloaded from the NCBI SRA database and mapped to the *atp6* and *ccmFc* gene sequences using BWA v0.7 [67]. Editing efficiency was estimated by calculating the proportion of cDNA reads that contained the edited nucleotide.

The 29 gene clusters in the plant mitogenomes described by Richardson et al. [97] were identified in the Ericales mitogenomes by manual inspection. Additional gene clusters were searched among *Primula* taxa through visual checking, as described by Kan et al. [25].

### Identification of conserved sequences and foreign-shared sequences

Each pair of *Primula* mitogenomes was aligned against the others to identify LCBs using the nucmer command in MUMMER4 [98] under the default parameters. The aligned areas with lengths < 2000 bp were removed. To calculate the interspecific nucleotide diversity of mitogenomes in plant genera, more than three mitogenomes (from different species) within each genus were considered and retrieved from GenBank (Table S8). The top three LCB and their CDSs within each genus were aligned using MAFFT v7.4 [95] and then concentrated using SequenceMatrix [99]. Nucleotide diversity was calculated using DnaSP v.6 [100].

Three *Primula* plastid genomes were assembled using GetOrganelle [101] and annotated using the GeSeq server [88]. Transfer events were identified by aligning each *Primula* mitogenome against the *P. palinuri* plastid genome (after removing one inverted repeat) using local BLASTN with a minimum length of 100 bp and an e-value of 1e-20. Putative MTPTs, including *atp1/atpA*, *rrn26/rrn23*, and *rrn18/rrn16,* were excluded because they simultaneously occurred in the mitogenome and plastid genome [20, 25, 66].

Two MTPTs longer than 500 bp (containing enough polymorphic variation for phylogenetic analysis) were extracted and validated using read mapping and PCR amplification. Each clean read was mapped against the 100 bp up- and downstream regions of MTPT in the mitogenome and its plastid genome counterpart for read mapping. MTPT7 and MTPT10 were aligned with the other 30 publicly available plastid genomes (Table S12) after validation within Primulaceae using MAFFT v7.4 [95]. Each aligned region was trimmed using Gblocks [102]. IQ-TREE v1.6.12 [103] was used to construct an ML tree with 5000 ultrafast bootstrapping replicates under the best model detected using ModelFinder [104]. Then, the GC content of the two sequences was calculated using an in-house Python3 script.

To account for gene transfer events between the mitogenome and nuclear genome, the nuclear-shared sequences were identified by aligning each *Primula* mitogenome against the *P. veris* nuclear genome (GCA_000788445.1) using BLASTN with a minimum length of 50 bp and an e-value of 1e-20. The nuclear-shared regions in other Ericales mitogenomes were also identified against their corresponding nuclear genomes. This reference nuclear genome information can be found in Table S13. The size information of mitogenomes, repeats, MTPTs, and NUMTs can be found in Table S10.

Repeat regions and MTPTs were also identified among Ericales mitogenomes. Pearson’s correlation was calculated between repeats, NUMTs, MTPTs, and mitogenome size in Ericales to reveal the underlying causes of mitogenome size variation [3].

### Phylogenetic analysis and identification of the contents of Ericales mitogenomes

The above eight publicly available Ericales mitogenomes and three *Primula* mitogenomes (representing Primulaceae, Ericaceae, Actinidiaceae, and Theaceae) were included in the phylogenetic analysis, with *Platycodon grandiflorus* A. DC. (NC_035958.1; Campanulaceae) and *Solanum lycopersicum* L. (NC_035963.1; Solanaceae) as outgroups. A total of 13 coding sequences (CDSs) among the mitogenomes were aligned with MAFFT v7.4 [95]. Conserved aligned regions were extracted with Gblocks [102] and concatenated with SequenceMatrix [99]. ML phylogenetic trees were built using IQ-TREE v1.6.12 [103] with 5000 ultrafast bootstrapping replicates under the GTR + G model. Bayesian inference was conducted using MrBayes v3.2 [105] under the GTR + G + F model. The Markov chain Monte Carlo (MCMC) algorithm was run for 2.0 × 10^7^ generations with four incrementally heated chains, starting from random trees and sampling one out of every 1000 generations. The stability of the Markov chain was ascertained by plotting likelihood values against the number of generations (effective sample size > 200) using Tracer v1.7 [106] and by splitting variances < 0.01. The burn-in fraction was set to 0.25, and the remaining trees were used to construct the 50%-majority rule consensus tree. Bayesian posterior probabilities were used to estimate support for each branch in the consensus tree.

## Supplementary Information

**Additional file 1: Figure S1.** Electrophoretic gel visualization of the amplified fragments of the three draft mitogenome assemblies and MTPTs. M1 is the DL2000 DNA marker, whereas M2 is the DL1000 DNA marker. The first two wells in each gel represent the corresponding primer pairs for MTPT7 and MTPT10 in each *Primula* mitogenome, whereas the other primer pairs were used for assembly validation in each draft mitogenome (primer details can be found in Table S11).

**Additional file 2: FigureS2.** The inferred secondary structure of the *atp6* gene among the Ericales mitogenomes. *Primula* contains three *Primula* mitogenomes; *Actinidia* contains two kiwifruit mitogenomes.

**Additional file 3: FigureS3.** The inferred secondary structure of the *ccmFc* gene among the Ericales mitogenomes. *Primula* contains three *Primula* mitogenomes; *Actinidia* contains two kiwifruit mitogenomes.

**Additional file 4: FigureS4.** Average coverage of MTPTs and their plastid counterparts.

**Additional file 5: Figure S5.** The three longest LCBs and their distribution in *Primula* taxa.

**Additional file 6: FigureS6.** Gene clusters of each Ericales mitogenome. The red cell indicates the existence of one specific gene cluster.

**Additional file 7: Figure S7.** The newly identified gene clusters of each Ericales mitogenome. The red cell indicates the existence of one specific gene cluster.

**Additional file 8: Figure S8.** Correlation of NUMT length and Ericales mitogenome size.

**Additional file 9: Figure S9.** Correlation of NUMTs, MTPTs, and repeat length and Ericales mitogenome size after removing outlier mitogenomes. (a) NUMT length and Ericales mitogenome size after removing the *Aegiceras corniculatum* mitogenome; (b) repeat length and Ericales mitogenome size after removing the kiwifruit mitogenomes; (c) MTPT length and Ericales mitogenome size after removing the *Rhododendron simsii* and *Monotropa hypopitys* mitogenomes.

**Additional file 10: Table S1**. Assembly information of three *Primula* taxa. **Table S2.** The number of protein-coding genes in the three *Primula* mitogenomes. **Table S3**. Tandem repeats in the *Primula smithiana* mitogenome. **Table S4**. Tandem repeats in the *Primula palinuri* mitogenome. **Table S5.** Tandem repeats in the *Primula valentiniana* mitogenome*.* **Table S6**. Information on MTPTs within the three *Primula* mitogenomes. **Table S7.** The shared nuclear sequences within the three *Primula* mitogenomes. **Table S8.** The nucleotide diversity of mitogenomes within different plant lineages. **Table S9.** The nucleotide diversity of mitogenomes among *Primula* taxa. **Table S10.** The general features of the 11 mitogenomes used for this study. **Table S11.** The primers used for *Primula palinuri*, *Primula smithiana*, and *Primula valentiniana*. **Table S12.** The 30 publicly available plastid genomes used for MTPT analysis. **Table S13.** The publicly available nuclear genomes used for NUMT analysis.

## Data Availability

The data of this study have been deposited in the NCBI with BioProject accession number PRJNA794031. The genome skimming sequencing reads can be found under the number SRR17422315-SRR17422317. Mitogenome assembly of *Primula* has been deposited to GenBank with accession numbers OM971881-OM971883. Plastid genomes of three *Primula* taxa have been deposited to GenBank with accession numbers OM313289-OM313291.

## Abbreviations

bp: base pair
CDS: Coding sequence
DNA: Deoxyribo Nucleic acid
GC: Guanine- cytosine
HGT: Horizontal gene transfer
HR: Homologous rearrangement
kb: kilobase
LCB: Locally collinear blocks
MTPT: Mitochondrial plastid DNA
ML: Maximum likelihood
mtDNA: mitochondrial DNA
NUMT: Nuclear mitochondrial DNA sequences
IGT: Intracellular gene transfer
ORF: Open reading frame
PCR: Polymerase chain reaction
RNA: Ribonucleic acid
atp1, atp4, atp6, atp8 and atp9: Genes for ATP synthase subunits 1, 4, 6, 8 and 9
ccmC, ccmC, ccmFc and ccmFn: Genes for cytochrome c biogenesis proteins B, C, Fc and Fn
cob: Gene for cytochrome b
cox1, cox2 and cox3: Genes for cytochrome c oxidase subunits 1, 2 and 3
matR: Gene for maturase
mttB: transport membrane protein B
nad1, nad2, nad3, nad4, nad4L, nad5, nad6, nad7 and nad9: Mitochondrial genes for NADH dehydrogenase subunits 1–7, 9 and 4 L
rpl2, rpl5 and rpl16: Genes for ribosomal proteins L2, L5 and L16
rps1, rps2, rps3, rps4, rps7, rps10, rps11, rps12, rps13, rps14 and rps19: Genes for ribosomal proteins S1, S2, S3, S4, S7, S10, S11, S12, S13, S14, and S19
sdh3 and sdh4: Genes for succinate dehydrogenase cytochrome subunits 3 and 4
trnL: tRNA gene for leucine
trnT: tRNA gene for threonine
trnS: tRNA gene for serine
Arg: Arginine
Cys: L-cysteine
His: Histidine
Pro: Proline
Ser: Serine
Tyr: Tyrosine
