# Supplementary material for: Comparative analyses of three complete Primula mitogenomes with insights into mitogenome size variation in Ericales
Source: BMC Genomics. 2022 Nov 24;23:770. doi: 10.1186/s12864-022-08983-x (PMC9686101; doi:10.1186/s12864-022-08983-x)
Supplement: Supplementary file 1 — Additional file 1: Figure S1. Electrophoretic gel visualization of the amplified fragments of the three draft mitogenome assemblies and MTPTs. M1 is the DL2000 DNA marker, whereas M2 is the DL1000 DNA marker. The first two wells in each gel represent the corresponding primer pairs for MTPT7 and MTPT10 in each Primula mitogenome, whereas the other primer pairs were used for assembly validation in each draft mitogenome (primer details can be found in Table S11). [file 12864_2022_8983_MOESM1_ESM.pdf]

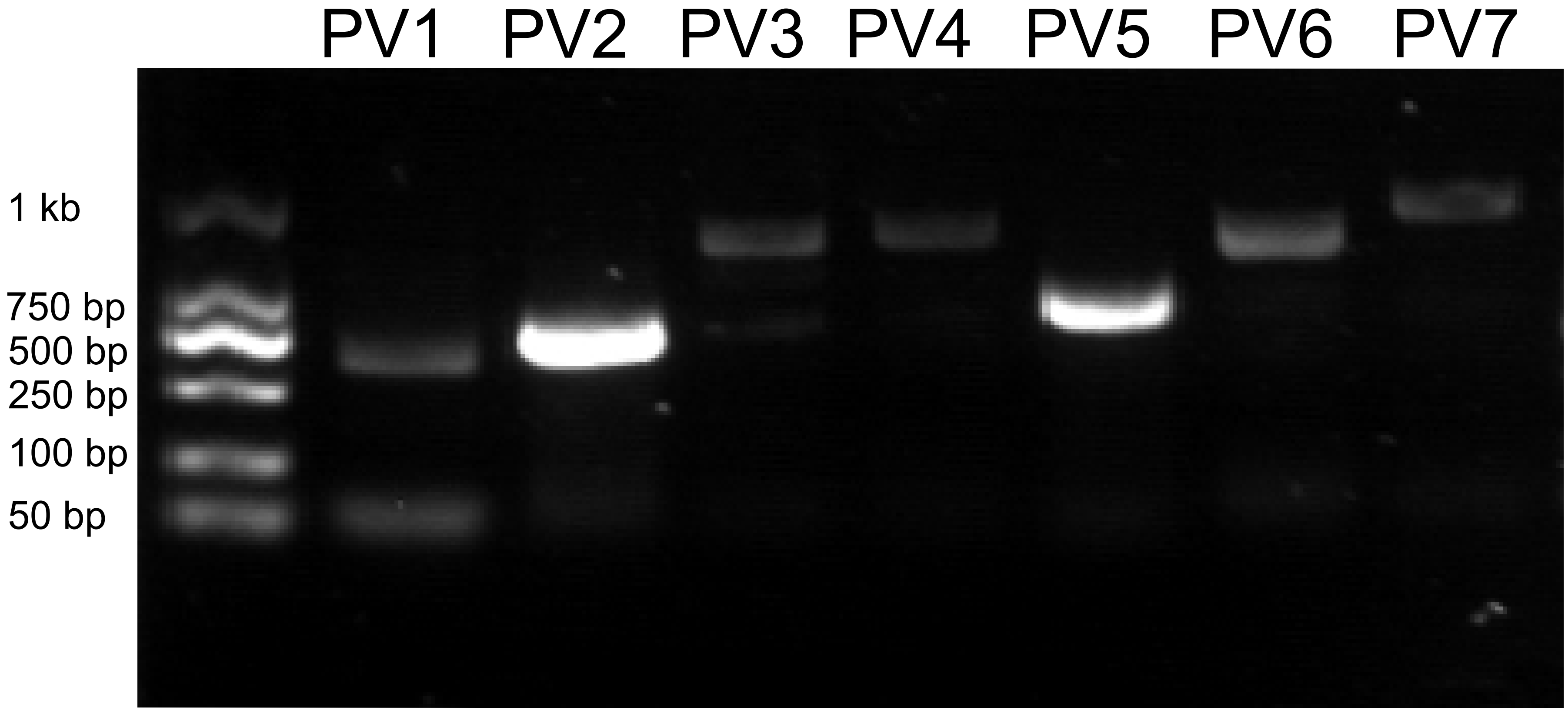

Primula valentiniana

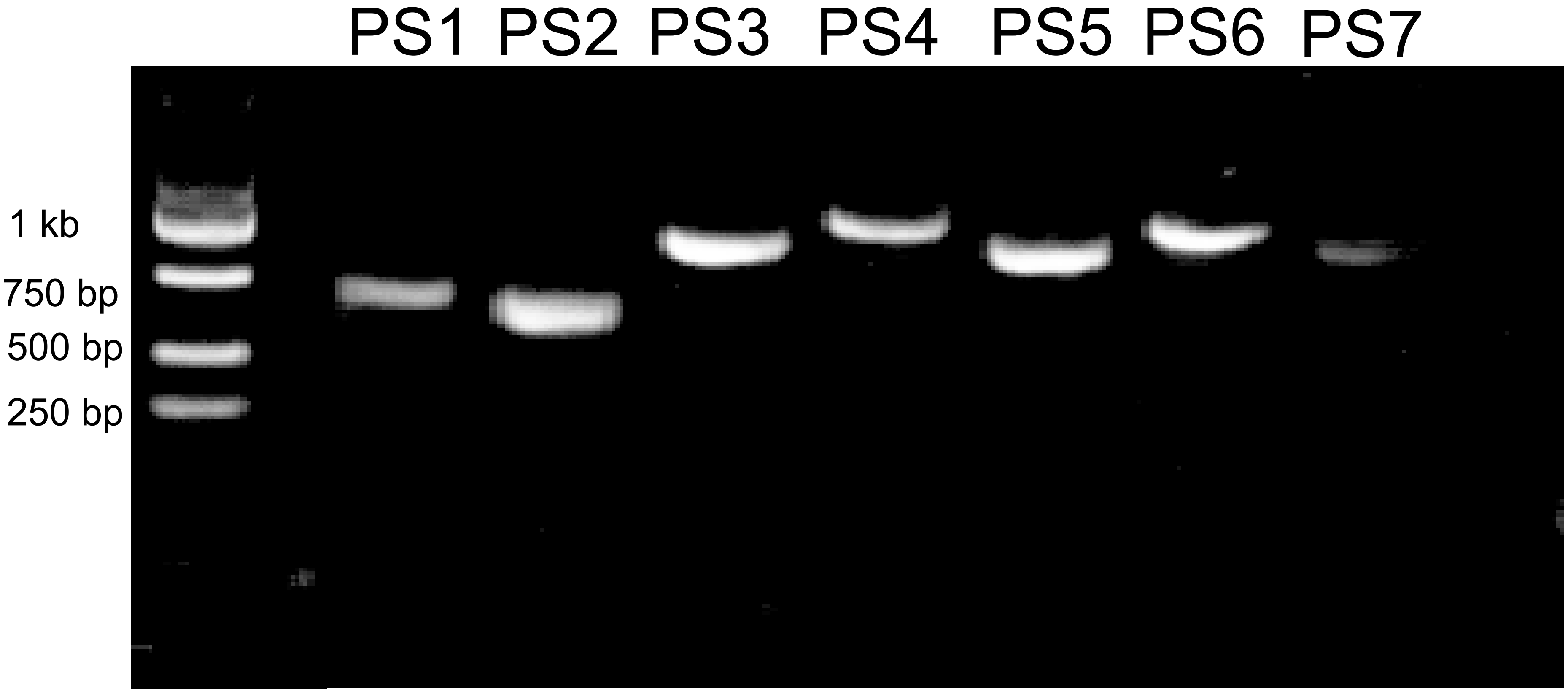

Primula smithiana

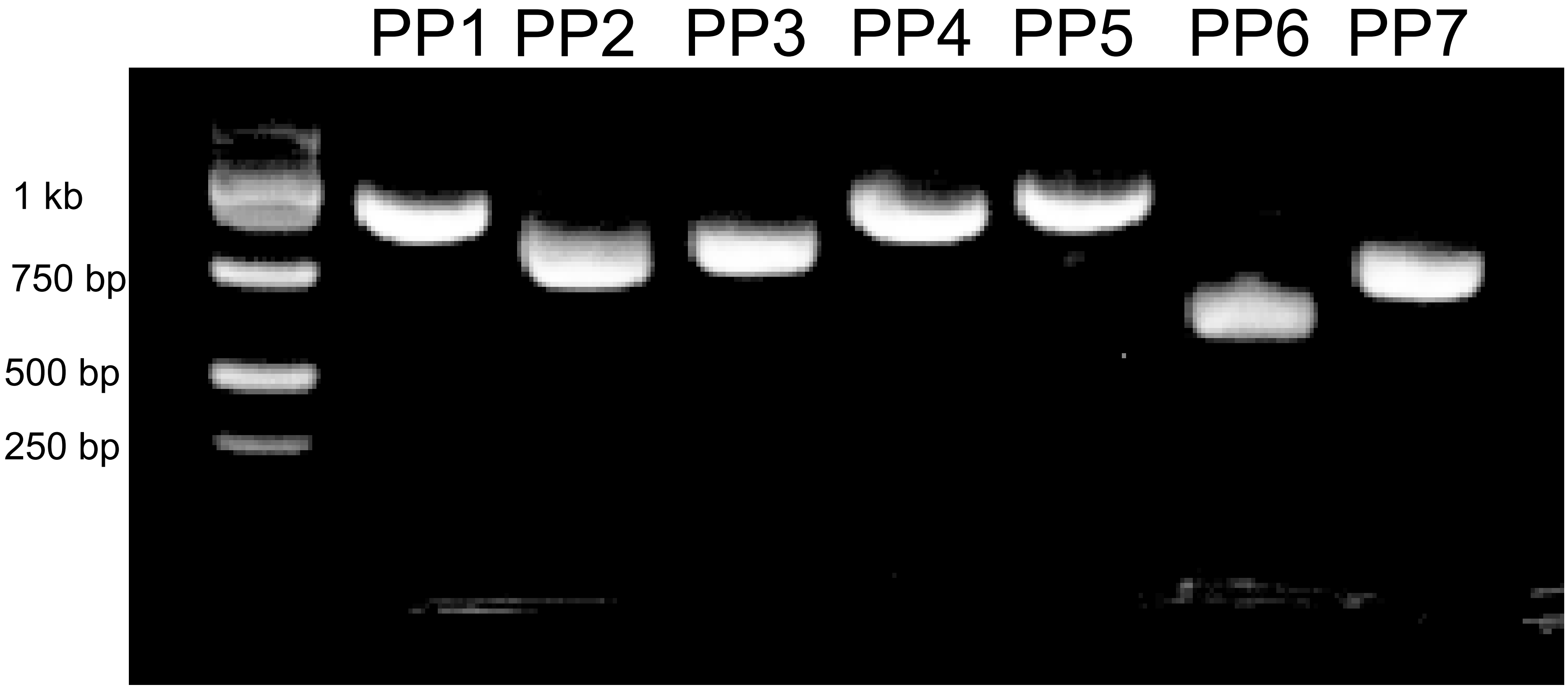

Primula palinuri
