# Supplementary figures and images for: Comparative analyses of three complete Primula mitogenomes with insights into mitogenome size variation in Ericales
Source: BMC Genomics. 2022 Nov 24;23:770. doi: 10.1186/s12864-022-08983-x (PMC9686101; doi:10.1186/s12864-022-08983-x)

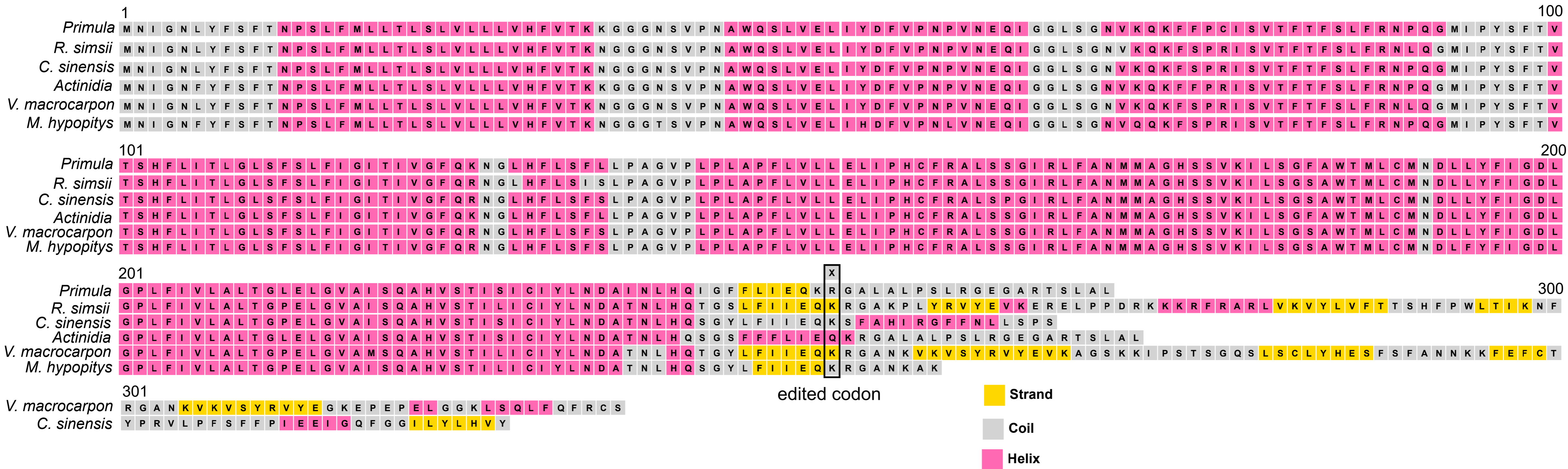

Supplement: Supplementary file 2 — Additional file 2: FigureS2. The inferred secondary structure of the atp6 gene among the Ericales mitogenomes. Primula contains three Primula mitogenomes; Actinidia contains two kiwifruit mitogenomes. [file 12864_2022_8983_MOESM2_ESM.pdf]

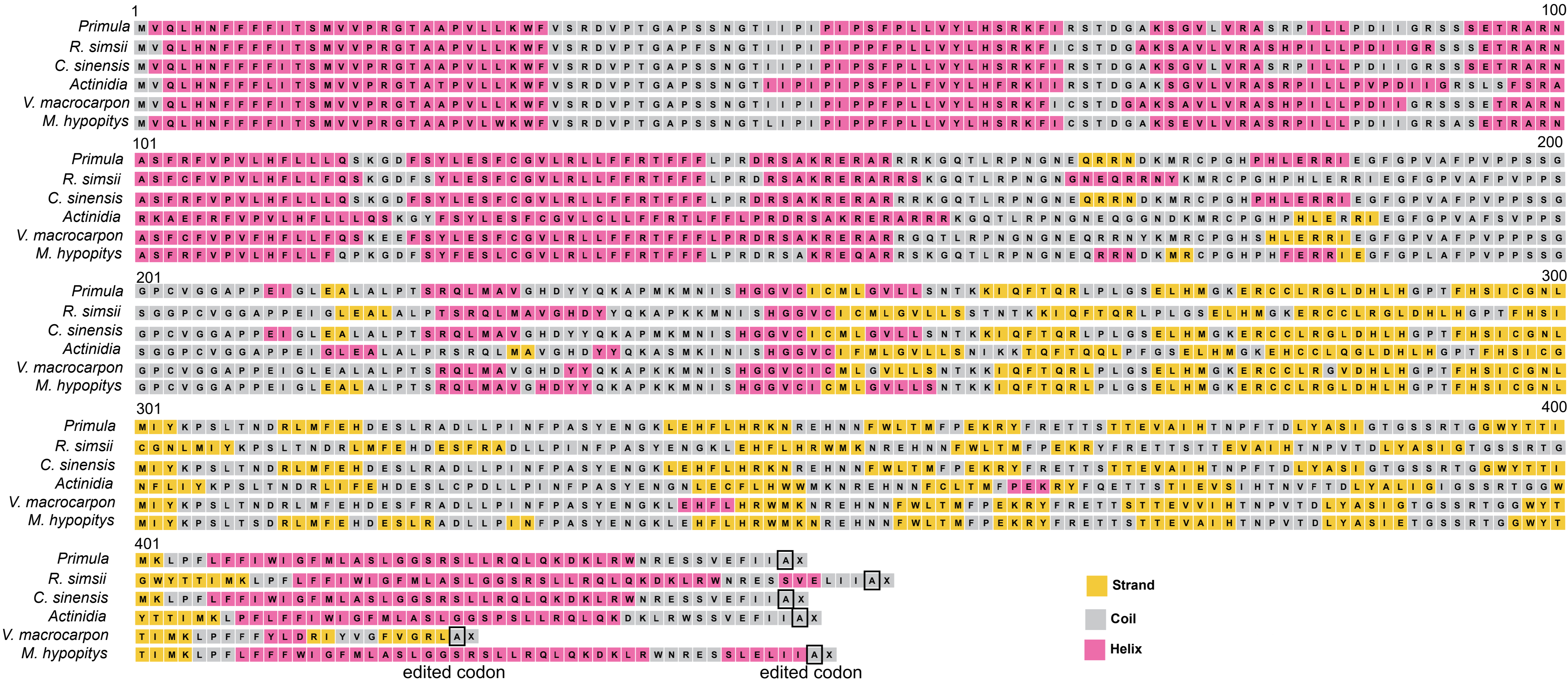

Supplement: Supplementary file 3 — Additional file 3: FigureS3. The inferred secondary structure of the ccmFc gene among the Ericales mitogenomes. Primula contains three Primula mitogenomes; Actinidia contains two kiwifruit mitogenomes. [file 12864_2022_8983_MOESM3_ESM.pdf]

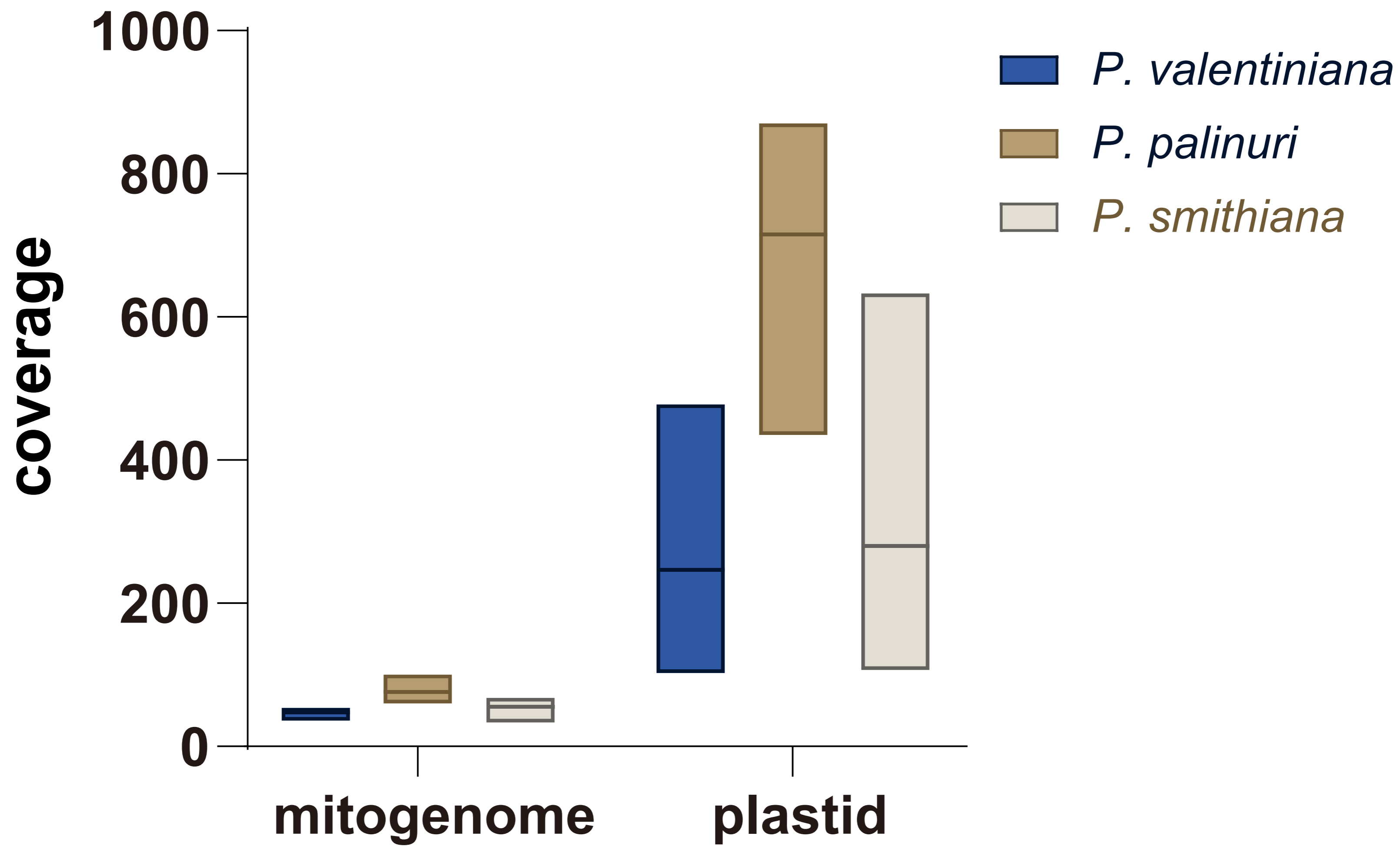

Supplement: Supplementary file 4 — Additional file 4: FigureS4. Average coverage of MTPTs and their plastid counterparts. [file 12864_2022_8983_MOESM4_ESM.pdf]

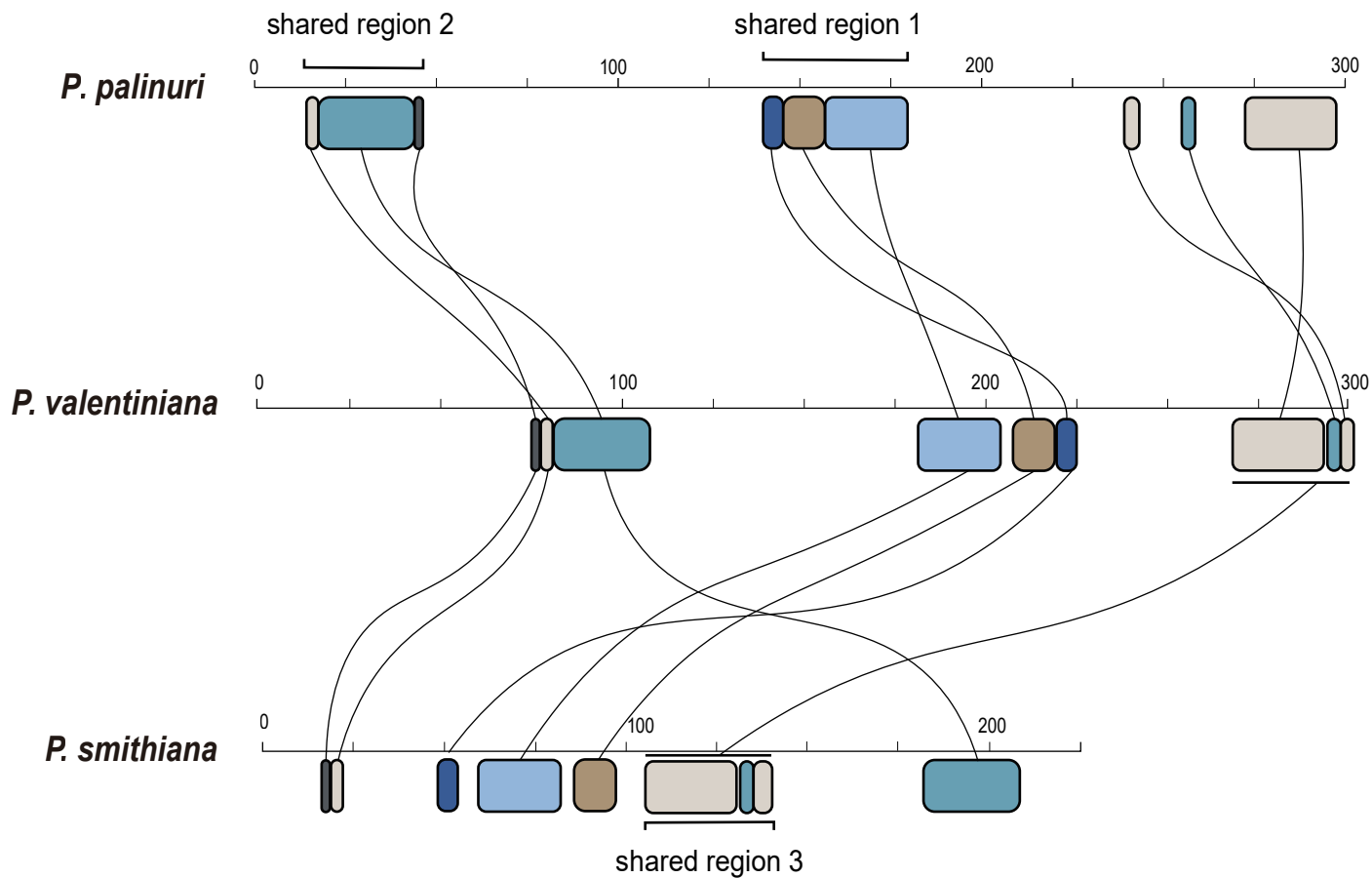

Supplement: Supplementary file 5 — Additional file 5: Figure S5. The three longest LCBs and their distribution in Primula taxa. [file 12864_2022_8983_MOESM5_ESM.pdf]

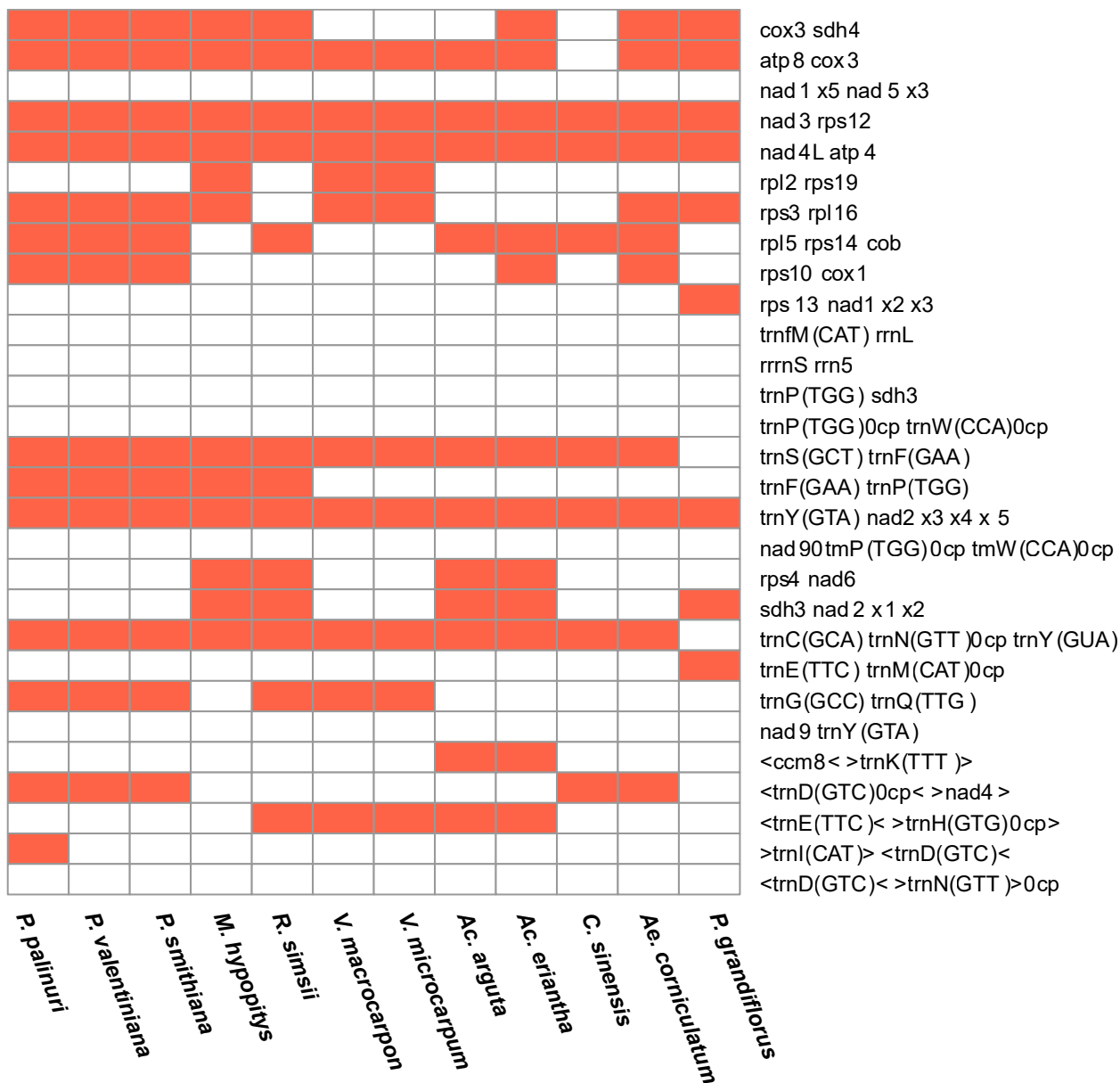

Supplement: Supplementary file 6 — Additional file 6: FigureS6. Gene clusters of each Ericales mitogenome. The red cell indicates the existence of one specific gene cluster. [file 12864_2022_8983_MOESM6_ESM.pdf]

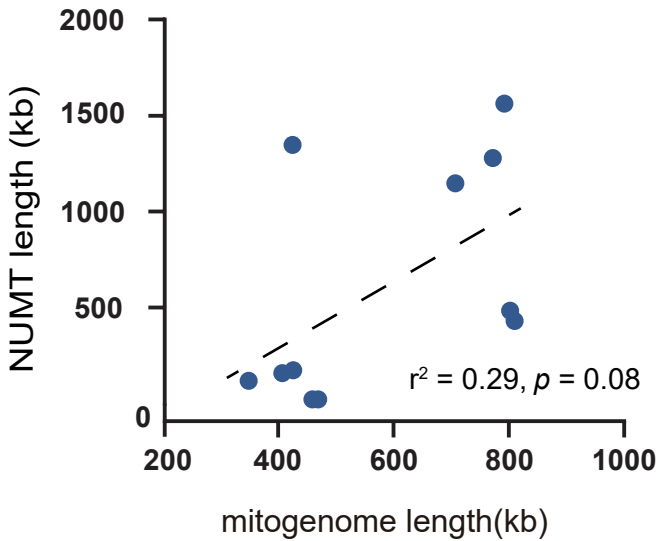

Supplement: Supplementary file 8 — Additional file 8: Figure S8. Correlation of NUMT length and Ericales mitogenome size. [file 12864_2022_8983_MOESM8_ESM.pdf]

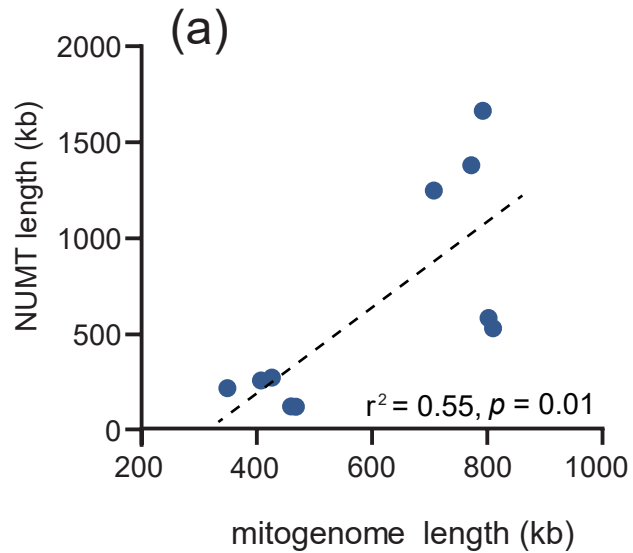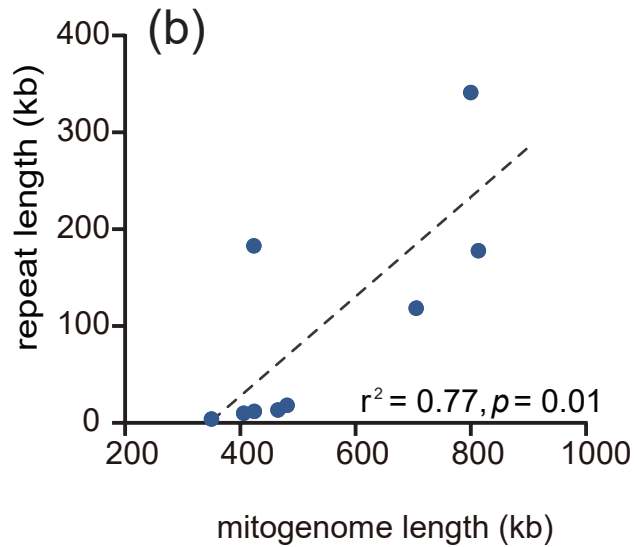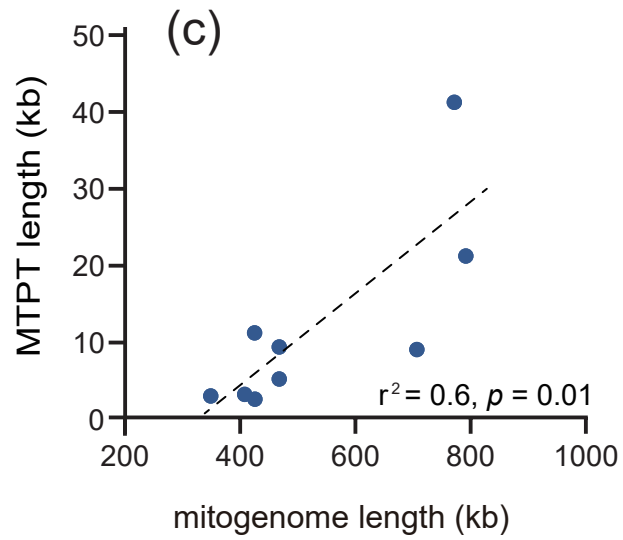

Supplement: Supplementary file 9 — Additional file 9: Figure S9. Correlation of NUMTs, MTPTs, and repeat length and Ericales mitogenome size after removing outlier mitogenomes. (a) NUMT length and Ericales mitogenome size after removing the Aegiceras corniculatum mitogenome; (b) repeat length and Ericales mitogenome size after removing the kiwifruit mitogenomes; (c) MTPT length and Ericales mitogenome size after removing the Rhododendron simsii and Monotropa hypopitys mitogenomes. [file 12864_2022_8983_MOESM9_ESM.pdf]
