# Supplementary material for: Comparative analyses of three complete Primula mitogenomes with insights into mitogenome size variation in Ericales
Source: BMC Genomics. 2022 Nov 24;23:770. doi: 10.1186/s12864-022-08983-x (PMC9686101; doi:10.1186/s12864-022-08983-x)
Supplement: Supplementary file 7 — Additional file 7: Figure S7. The newly identified gene clusters of each Ericales mitogenome. The red cell indicates the existence of one specific gene cluster. [file 12864_2022_8983_MOESM7_ESM.pdf]

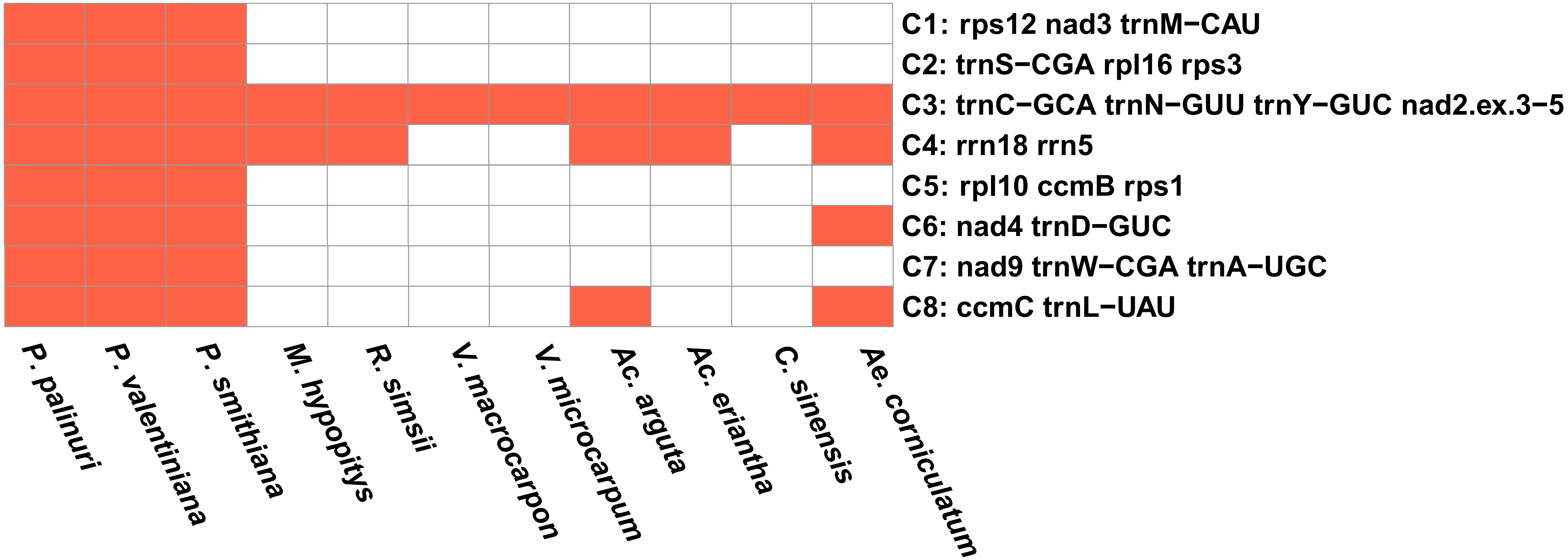
C1: rps12 nad3 trnM−CAU
C2: trnS−CGA rpl16 rps3
C3: trnC−GCA trnN−GUU trnY−GUC nad2.ex.3−5
C4: rrn18 rrn5
C5: rpl10 ccmB rps1
C6: nad4 trnD−GUC
C7: nad9 trnW−CGA trnA−UGC
C8: ccmC trnL−UAU
P. palinuri
P. valentiniana
P. smithiana
M. hypopitys
R. simsii
V. macrocarpon
V. microcarpum
Ac. arguta
Ac. eriantha
C. sinensis
Ae. corniculatum
